# Heterologous expression of *Arabidopsis thaliana rty* gene in strawberry (*Fragaria* × *ananassa* Duch.) improves drought tolerance

**DOI:** 10.1186/s12870-021-02839-4

**Published:** 2021-01-21

**Authors:** Maofu Li, Yuan Yang, Ali Raza, Shanshan Yin, Hua Wang, Yuntao Zhang, Jing Dong, Guixia Wang, Chuanfei Zhong, Hong Zhang, Jiashen Liu, Wanmei Jin

**Affiliations:** 1 Beijing Academy of Forestry and Pomology Sciences, Beijing Academy of Agriculture and Forestry Sciences, Beijing, 100093 P. R. China; 2 Key Laboratory of Biology and Genetic Improvement of Horticultural Crops (North China), Ministry of Agriculture, Beijing, 100093 P. R. China; 3 Beijing Engineering Research Center for Deciduous Fruit Trees, Beijing, 100093 P. R. China; 4 Key Laboratory of Biology and Genetic Improvement of Oil Crops, Oil Crops Research Institute, Chinese Academy of Agricultural Sciences (CAAS), Wuhan, 430062 P. R. China

**Keywords:** ABA, *Arabidopsis thaliana*, Drought stress, Heterologous expression, Stomatal closure, Strawberry

## Abstract

**Background:**

Strawberry (*Fragaria* × *ananassa* Duch.) is an important fruit crop worldwide. It was particularly sensitive to drought stress because of their fibrous and shallow root systems. Mutant *rty* of *Arabidopsis thaliana* ROOTY (*RTY*) results in increased endogenous auxin levels, more roots, and shoot growth. It is still unclear whether the *rty* gene improves stress tolerance in strawberry.

**Results:**

*rty* gene was isolated from *Arabidopsis thaliana* and placed under the control of the cauliflower mosaic virus (CaMV) 35S promoter in the pBI121-rty binary vector carrying the selectable marker of neomycin phosphotransferase II (*NPT* II). Seven transgenic lines were confirmed by PCR and western blot analysis. Accumulations of IAA and ABA were significantly increased in the transgenic plants. The endogenous IAA contents were 46.5 ng g^− 1^ and 66.0 ng g^− 1^in control and transgenic plants respectively. The endogenous ABA contents in the control plant were 236.3 ng g^− 1^ and in transgenic plants were 543.8 ng g^− 1^. The production of adventitious roots and trichomes were enhanced in the transgenic plants. Furthermore, transcript levels of the genes including IAA and ABA biosynthetic, and stress-responsive genes, were higher in the transgenic plants than in the control plants under drought conditions. Water use efficiency and a reduced water loss rate were enhanced in the transgenic strawberry plants. Additionally, peroxidase and catalase activities were significantly higher in the transgenic plants than in the control plants. The experiment results revealed a novel function for *rty* related to ABA and drought responses.

**Conclusions:**

The *rty* gene improved hormone-mediated drought tolerance in transgenic strawberry. The heterologous expression of *rty* in strawberry improved drought tolerance by promoting auxin and ABA accumulation. These phytohormones together brought about various physiological changes that improved drought tolerance via increased root production, trichome density, and stomatal closure. Our results suggested that a transgenic approach can be used to overcome the inherent trade-off between plant growth and drought tolerance by enhancing water use efficiency and reducing water loss rate under water shortage conditions.

**Supplementary Information:**

The online version contains supplementary material available at 10.1186/s12870-021-02839-4.

## Background

Plant hormones regulate myriad aspects of plant growth and development. Auxins are plant growth hormones that cause rapid increases in plant cell wall extensibility, alter ion flux at the plasma membrane, and cause specific changes in gene expression [1]. Indole-3-acetic acid (IAA) is a classic plant auxin that regulates embryogenesis, tropic growth, leaf formation, stem elongation, fruit development, and root formation [2–5].

A mutational analysis of *Arabidopsis thaliana* revealed the importance of auxins in plant growth and development. The *aberrant lateral root formation1* (*alf1*), *superroot1* (*sur1*), and *ROOTY* mutant (*rty*) accumulate increased levels of endogenous auxins, which lead to the development of an increased number of roots [6–9]. These observations are consistent with the results of previous studies that established that auxins promote lateral root development [6–8, 10, 11]. In *A. thaliana*, the *rooty* (*rty*) mutation was shown to be allelic to both *superroot1* (*sur1*) and the ethylene-response mutant *hookless3* (*hls3*) [6–8, 12]. *rty* encodes an aminotransferase or a C-S lyase that catalyzes IAA biosynthesis and may influence auxin transport and increase levels of both free and conjugated IAA [7–9]. The gene expression of *rty* may clarify its direct or indirect regulation of auxin concentrations and elucidate the effects of this locus on plant growth and development, including the formation of adventitious and lateral roots, as well as the controlled expansion of the shoot [8].

The aminotransferase encoded by *rty* is also responsive to abscisic acid (ABA), which exhibits increased levels due to crosstalk between the auxin and ABA biosynthesis and metabolism pathways [9]. ABA and IAA functionally interact in roots as regulators of growth, development, and tropisms [9, 12–15].

ABA is a classic stress-associated plant hormone that improves tolerance to abiotic stresses [16]. Drought stress is among the most destructive abiotic stresses in the agricultural industry. Plants often experience seasonal water stress due to variable rainfall [17]. ABA is an important modulator of the drought stress response in plants due to its effects on guard cell development, stomatal aperture closure, and the expression of specific genes associated with drought tolerance [18–21]. The physiological response of plants to drought directly affects growth, productivity, and survival under water shortage conditions [22–24]. Despite the importance of auxin and ABA in the plant’s response to drought stress, little is known of how the *rty*-encoded aminotransferase influences ABA accumulation, improves drought tolerance, or how variation in ABA accumulation may contribute to environmental adaptation.

Strawberry (*Fragaria* × *ananassa* Duch.), which is a flavorful and popular fruit crop, is an important source of various minerals and vitamins in the world [25, 26]. The strawberry genome carries eight sets of chromosomes (2n = 8x = 56), which are derived from four diploid ancestors [27]. Because of the superior fruit quality of this hybrid subspecies, it was rapidly distributed around the wide [28]. Cultivated strawberry has grown to be one of the most important fruit crop plants worldwide, with a total annual production of over 8 million tons [29]. Due to their fibrous and shallow root systems, strawberry plants are particularly sensitive to drought stress. It is challenging to cultivate strawberries in drought-prone regions.

In this study, we analyzed the effect of *rty* on adventitious root development in strawberry. Our data revealed a novel function for *rty* related to ABA and drought responses, indicating that this gene has roles beyond the regulation of plant growth and development. The heterologous expression of *rty* enhances plant tolerance to drought stress via the ABA-mediated regulation of stomatal closure in strawberry. These results have potential significance for the cultivation of strawberries in drought-prone regions.

## Results

### Constitutive heterologous expression of *rty* increases the ABA and IAA contents

**Fig. 1:**
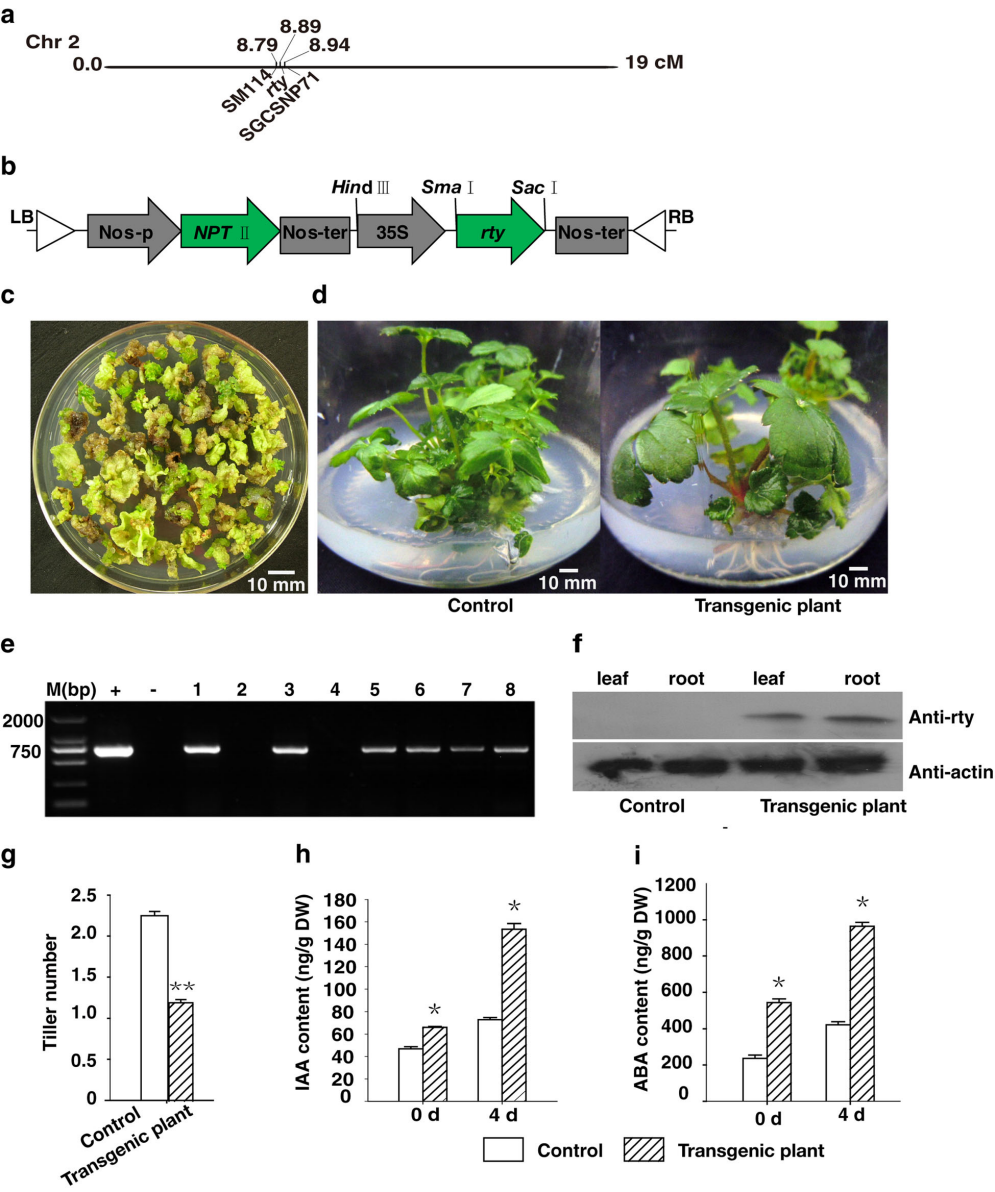
Heterologous expression of *rty* in strawberry increased endogenous IAA and ABA contents. **a** Arabidopsis *rty* is located at position 8.89 cM of chromosome 2. **b** Schematic diagram of the pBI121-*rty* vector construct used to transform strawberry. **c** Resistant shoots were detected on the medium MS + 6 - BA 3.0 mg L^− 1^ + 2, 4 - D 0.1 mg L^− 1^ + kanamycin 5 mg L^− 1^. **d** Comparison of the control and transgenic plants heterologously expressing *rty*. **e** Transgenic plants were analyzed by PCR. 1–8 represented various transgenic plants, whereas + (positive control) represented the pBI121-*rty* plasmid and - represented the untransformed plant (negative control). M (bp) represented DNA marker (base pair). Full-length gels image is presented in Additional file 2. **f** Transgenic plants were further analyzed by western blot with anti-rty and anti-actin. Full-length blots images are presented in Additional files 3 and 4. **g** Tiller number of the control and transgenic plants. Data are presented as the mean ± SD (*n* = 15) (^**^P < 0.01, Student’s t test). The endogenous IAA **h** and ABA (**i**) contents of the control and transgenic plants were analyzed at days 0 and 4. One month old control and transgenic plants were inoculated on MS medium 4 days with no exogenous growth regulators. The first day were defined the 0 day after inoculating on the medium. About 0.4 cm basic segments of control and transgenic plant at 0 day and 4 days were sampled. Data were presented as the mean ± SD (*n* = 3) (^*^P < 0.05, Student’s t test)

**Fig. 2:**
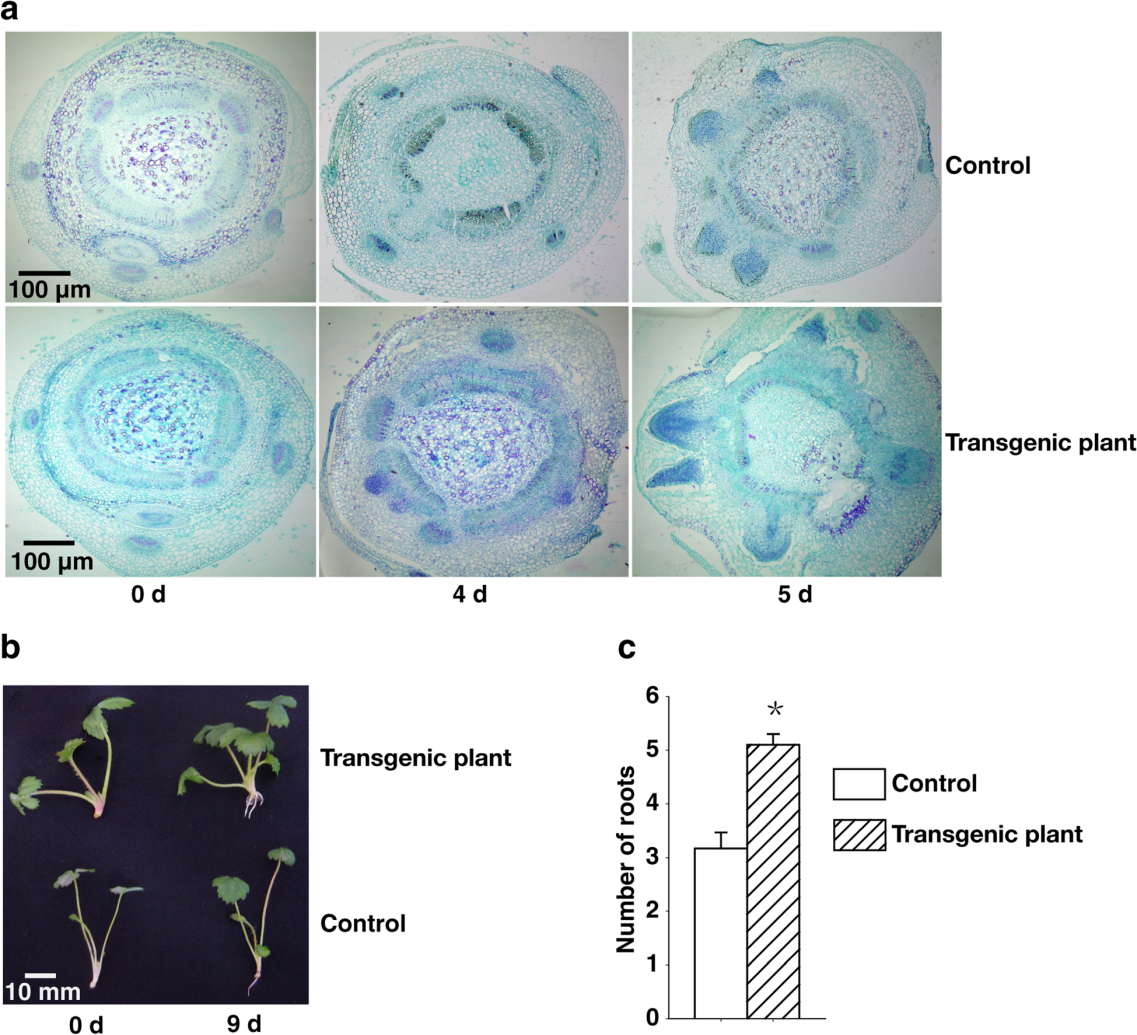
High endogenous auxin contents led to early root development and increased numbers of roots. **a** Paraffin sections were prepared for control and transgenic samples collected on days 0, 4, and 5 for an analysis of adventitious root formation by histological observation. **b** The control and transgenic plants heterologously expressing were grown on MS medium with no exogenous growth regulators and were analyzed on days 0 and 9. **c** Comparison of the number of roots in the control and transgenic plants at ninth days. Data were presented as the mean ± SD (n = 15) (^*^P < 0.05, Student’s t test)

In *A. thaliana*, *rty* is a critical regulator of endogenous auxin concentration, with consequences for normal growth and development [6–8]. The *RTY* gene is located at 8.89 cM between marker SM114 (8.79 cM) and SGCSNP71 (8.94 cM) on chromosome 2 (Fig. 1a). To determine the regulatory effect of *rty* on adventitious root development in strawberry, *rty* was introduced into strawberry via *Agrobacterium tumefaciens* (Fig. 1b). Putatively transformed shoots were identified by screening the culture (Fig. 1c). Seven transgenic lines were confirmed by PCR and western blot analysis (Fig. 1e, f, Additional files 2, 3, 4). The transgenic strawberry plants exhibited strong growth, including broad leaves (Fig. 1d), an increased number of roots (Fig. 2b, c), and leaf trichomes (Fig. 5b, d), but showed reduced tiller number (Fig. 1d, g) compared to the control plants.

The *rty* gene encodes either a transaminase or a C-S lyase involved in IAA biosynthesis [7–9]. *rty* appears to be critical for regulating IAA concentrations [6, 7, 9]. Thus, we expected the heterologous expression of *rty* in strawberry to result in IAA accumulation. Therefore, we assayed the endogenous IAA contents on days 0 and 4 in control and *rty* transgenic plants cultured on MS medium with no exogenous growth regulators. The concentrations of IAA in the control plants were 46.5 ng g^− 1^ on day 0 and 72.7 ng g^− 1^ on day 4, but in transgenic plants were 66.0 and 155.3 ng g^− 1^ respectively. Additionally, the IAA concentrations in the transgenic plants significantly increased between the two analyzed time-points, whereas the IAA concentration in the control plants only slightly increased (Fig. 1h). Thus, quantification of IAA concentrations revealed that heterologous expression of *rty* significantly enhanced the accumulation of IAA in the transgenic plants on days 0 and 4.

To clarify whether *rty* increased ABA levels due to crosstalk between the auxin and ABA biosynthesis and metabolism pathways [9], we next tested the endogenous ABA concentrations. The concentrations of ABA in the control plants were 236.3 ng g^− 1^ on day 0 and 421.7 ng g^− 1^ on day 4, but in transgenic plants were 543.8 and 963.4 ng g^− 1^, respectively. The ABA levels exhibited a similar trend as IAA, with endogenous ABA concentrations being significantly higher in the transgenic plants than in the control plants (Fig. 1i). These data indicate that the heterologous expression of *rty* in the transgenic strawberry plants stimulated the accumulation of large amounts of IAA and ABA.

### High endogenous auxin contents lead to early root development

Endogenous IAA regulates auxin-dependent developmental processes in plants, including adventitious root formation [6–8]. To assess the effects of the increased IAA concentration in the transgenic strawberry plants on root development, we examined the histology of the roots of control and transgenic plants on MS medium with no exogenous growth regulators. The roots exhibited a developmental pattern common among woody perennials. The meristematic tissues, including the exodermis, cortex, and stele, remained undifferentiated on the first day. There were no differences on day 0. Differences in root development between the control and transgenic plants were apparent on day 4. Specifically, in the transgenic plants at this stage, the arched nature of the xylem poles was lost, the periderm formed from the outer layers of the pericycle, and the outer cell layers containing the exodermis, cortex, and endodermis began to break down and rupture, but these phenomena were only observed on day 5 in control plants.

In the control plants on day 4, the primary xylem, primary phloem, and endodermis began to differentiate, and the vascular cambium formed and gave rise to secondary xylem and phloem tissues. Whereas the roots of the transgenic plants were intact on day 5, in control plants, the periderm had formed and the exodermis, cortex, and endodermis had ruptured by this time point (Fig. 2a). On day 9, most transgenic plants had 3–5 roots, whereas the control plants had 1–3 roots (Fig. 2b, c). These results suggest that the high IAA concentrations of the transgenic plants induced early root development and increased the number of roots.

### Heterologous expression of *rty* confers drought tolerance to strawberry

**Fig. 3:**
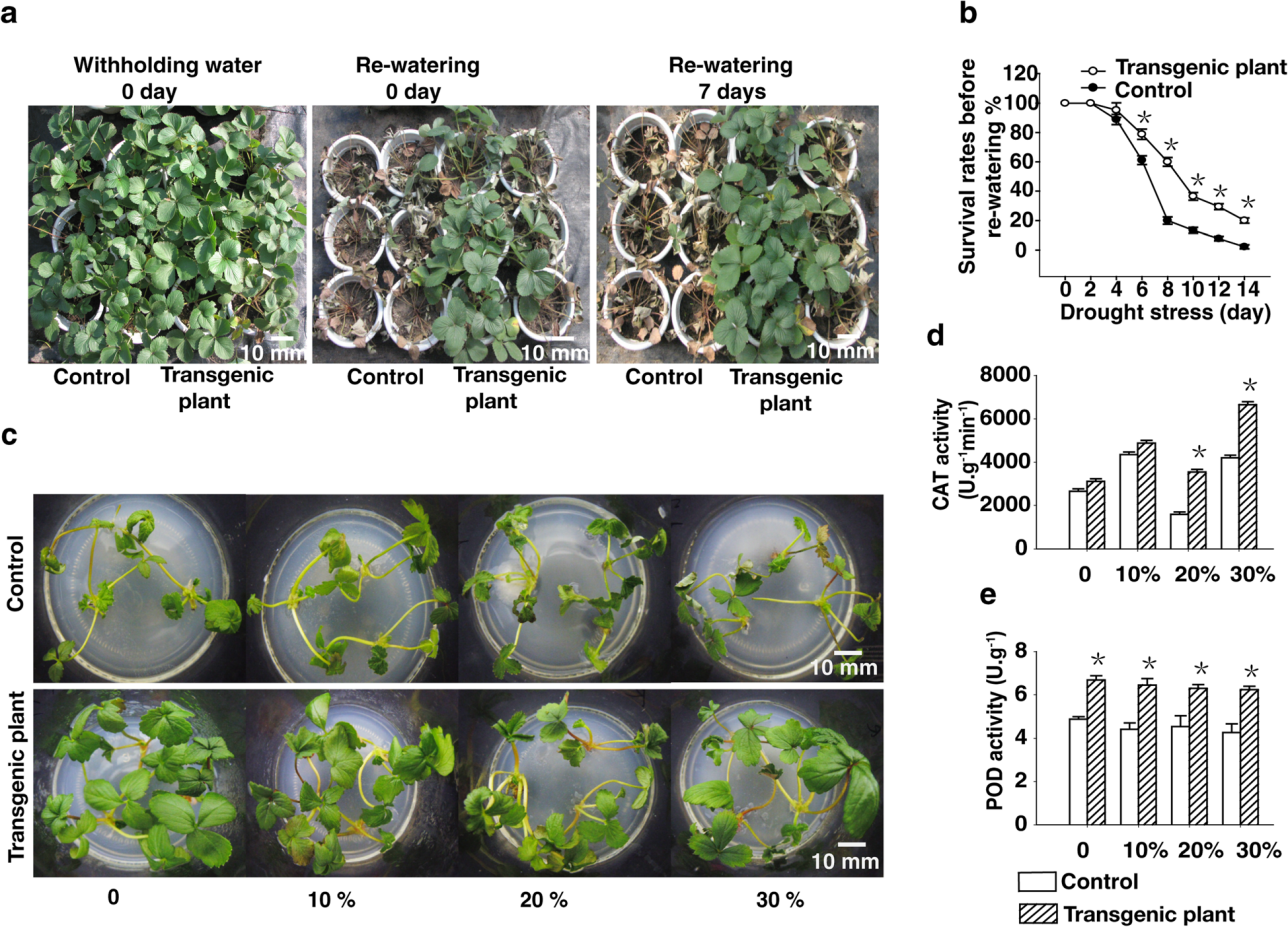
Heterologous expression *rty* in strawberry improved drought tolerance. **a** Control and transgenic strawberry plants were subjected to drought stress for by withholding water for 0 day. **b** Control and transgenic strawberry plants were subjected to drought stress by withholding water for 14 days. Survival rates were calculated on the rewatering day. **c** The 1 month old control and transgenic plants were cultured on subculture medium (MS + 6 - BA 0.2 mg L^− 1^ + IBA 0.1 mg L^− 1^) for 48 h on medium containing 0, 10, 20%, or 30% PEG 8000. **d** and **e** Antioxidant enzyme activities of the control and transgenic plants were analysis under the PEG 8000 treatment conditions. Data were presented as the mean ± SD (n = 3) (^*^P < 0.05, Student’s t test)

ABA accumulation in plants is expected to induce many drought-resistance mechanisms [21, 30, 31]. To clarify how the increased accumulation of ABA in the transgenic plants enhanced drought tolerance, we compared the effects of a drought treatment on the control and transgenic plants. Specifically, we grew the control and transgenic plants for 2 months in pots and then induced drought conditions by withholding water for 2 weeks. The plants were then rewatered and their growth was monitored for 1 week. The 14-day drought treatment resulted in curled and severely wilted leaves in the control plants, with many leaves withering and falling off the plants. By contrast, the leaves of the transgenic plants were less affected by the exposure to drought stress, with only a few leaves were curled, wilted, or withered (Fig. 3a). Additionally, 80% of the transgenic plants and 2% of the control plants survived the 14-day drought treatment (Fig. 3b). These results suggest that the enhanced drought tolerance of the transgenic plants was likely mediated by an ABA-dependent pathway.

Simultaneously, we measured the soil relative water content during drought stress. Whereas the relative soil water content was 100% in the control and transgenic plants on 0 d (i.e., the day of saturation with water), it was decreased from 19% on day 6 to 5.3% on day 14 in control, but decreased from 20% on day 6 to 6.6% on day14 in transgenic plants (Additional file 5). These data showed that the soil of transgenic plants was wetter by reducing transpiration.

To further characterize the drought tolerance of the transgenic plants, 30-day- old control and transgenic plants on subculture medium were treated with various PEG concentrations (0, 10, 20, and 30%) to simulate drought conditions. After 2 days of treatment with 0% PEG, the transgenic plants displayed no obvious differences from the untransformed control plants. However, after 2 days of treatment, leaves of the control plants began to roll and wilt in response to the 10% PEG treatment, with the rolling and wilting increasing in severity as the concentration of PEG increased to 30%. These symptoms were most severe for the 30% PEG treatment. By contrast, the leaves of the transgenic plants only began to roll and wilt in response to the 20% PEG treatment. The transgenic plants displayed less rolling and wilting than the control plants, even under 30% PEG conditions (Fig. 3c).

The ability of the control and transgenic plants treated with PEG to scavenge reactive oxygen species (ROS) was assessed by examining the activities of two key antioxidant enzymes (POD and CAT), which are known ROS scavengers. CAT activity was considerably higher in the transgenic plants than in the control plants. Additionally, the PEG treatment significantly enhanced the POD activity of the transgenic plants, ultimately resulting in significantly higher POD activity in the transgenic than control plants (Fig. 3d, e).

To explore the molecular mechanisms underlying the increased drought tolerance of the transgenic plants heterologously expressing *rty*, we analyzed the expression of stress-responsive genes during the PEG treatment using an RT-qPCR analysis. Specifically, we analyzed the expression of the following drought-responsive genes, involved in ABA biosynthesis, catabolism, transport, and signaling: *NCED3* (nine cis-epoxycarotenoid dioxygenase 3) [32], *ABI1* (ABA insensitive 1) [33], *RD29A* (responsive to dehydration 29) [34], *DREB2A* (dehydration responsive element-binding protein 2A) [35], and *PP2C* (type-2C protein phosphatase) [36]. The expression of *RD29A* (a stress-responsive marker), and *DREB2A* (a regulator of many water stress-inducible genes) during PEG treatment was upregulated to a greater extent in the transgenic plants than in the control plants (Additional file 6). The stress-responsive genes were likewise more responsive to drought stress in the transgenic plants than in the control plants, suggesting that stress signals are somehow amplified in *rty*, triggering a stronger drought response (Additional file 6). These results suggest that heterologous expression of *rty* in strawberry may decrease ROS accumulation by enhancing antioxidant enzyme activities.

### The water loss rate is lower in the transgenic plants than in the wild-type plants

**Fig. 4:**
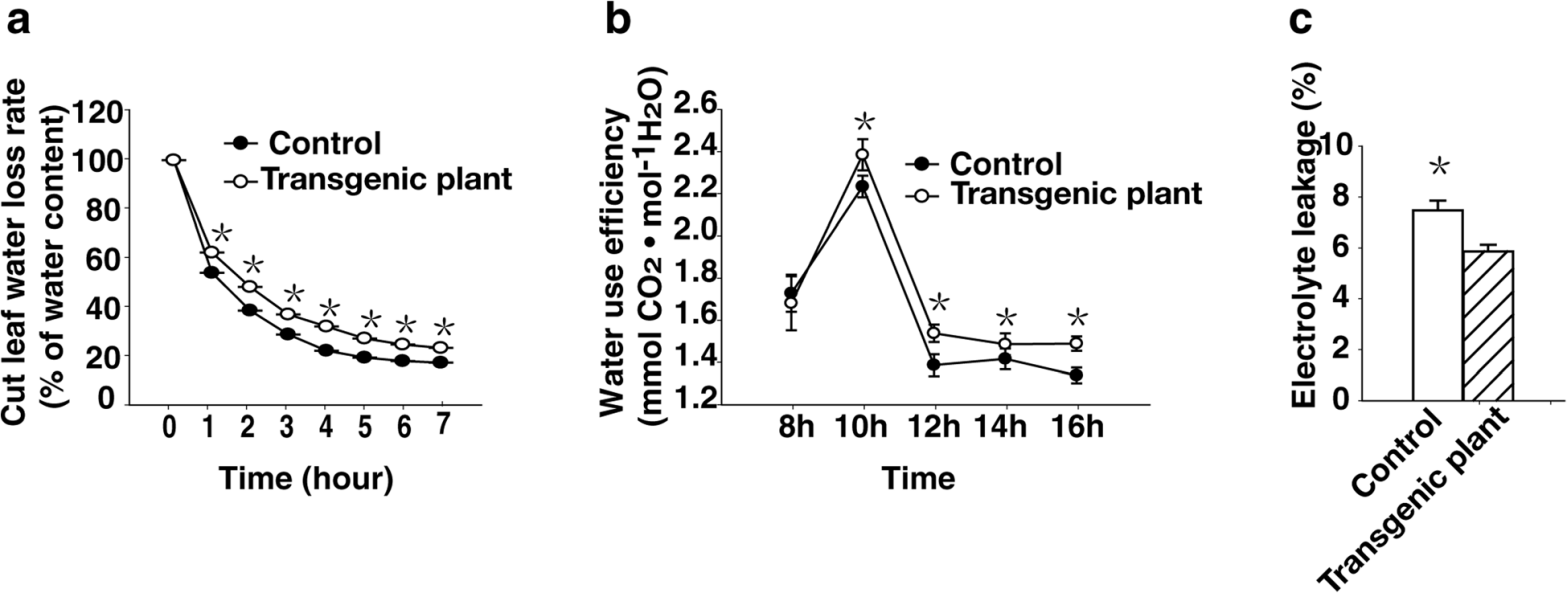
Heterologous expression of *rty* in transgenic strawberry plants resulted in reduced leaf water loss and electrolyte leakage, but increased water use efficiency. **a** Water loss rate of leaves were excised from the control and transgenic strawberry plants from 0 to 7 h (from am 8:00 to pm 3:00). **b** Water use efficiency of the control and transgenic plants from 8:00 to 16:00. Two-month-old plants were analyzed from pots in greenhouse. **c** Electrolyte leakage of the control and transgenic strawberry plants. Data were presented as the mean ± SD (n = 3) (^*^P < 0.05, Student’s t test)

To investigate the effects of heterologous expression of *rty* on the physiological status of strawberry plants, we analyzed the water loss rate, water use efficiency, and electrolyte leakage of the transgenic and control plants. The water loss rate of leaves of one-month-old plants at same position excised from three transgenic plants was lower than that of leaves from the control plants (Fig. 4a). The water use efficiency of leaves from two-month-old transgenic plants grown in pots in the greenhouse was higher than that of the control plants (Fig. 4b). Moreover, the electrolyte leakage from fresh leaves was lower in transgenic plants than in the control plants (Fig. 4c), likely due to the transgenic plants having less cell membrane damage. The differences in the water loss rate, electrolyte leakage, and water use efficiency between the control and transgenic plants may contribute to the improved drought tolerance of the transgenic plants heterologously expressing *rty*.

### Transgenic plants heterologously expressing *rty* have increased ABA-induced stomatal closure

**Fig. 5:**
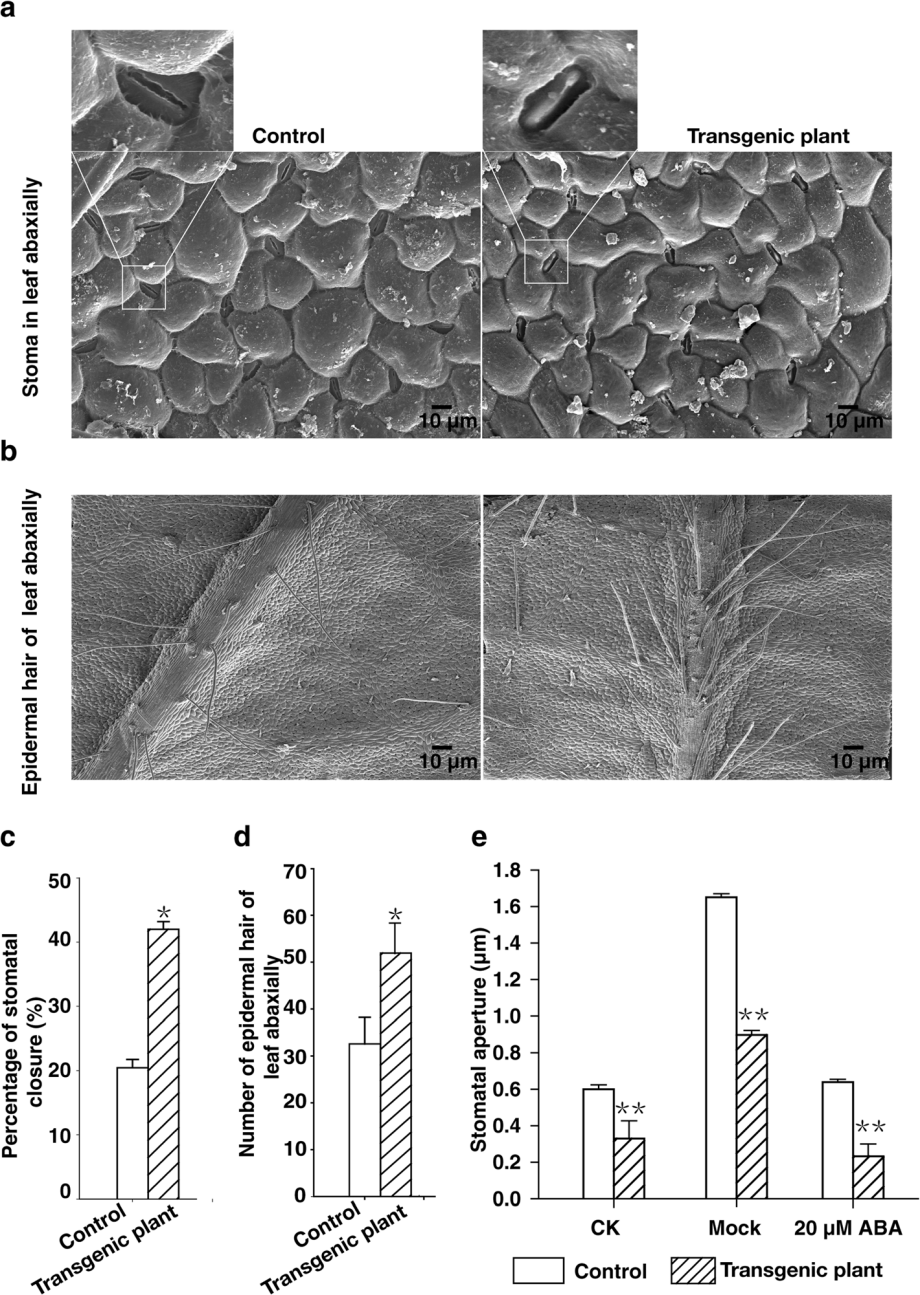
Heterologous expression of *rty* in strawberry promoted ABA-induced stomatal closure and stimulated the production of trichomes on the abaxial leaf surface. **a** Stomatal closure of control and transgenic plants at noon based on a scanning electron microscopy analysis. Images were enlargements of the boxed regions in (**a**). **b** Trichomes on the abaxial side of leaf surface between the control strawberry plants and transgenic plants. **c** and **d** Percentage of stomata closed and total number per unit area given epidermal trichomes on the abaxial surface of leaves from the control and transgenic plants based on a scanning electron microscopy analysis. Data were presented as the mean ± SD (n = 15) (^*^P < 0.05, Student’s t test). **e** ABA-induced stomatal closure. CK: not treated with stomatal opening solution (50 mmol L^− 1^ KCl, 10 mmol L^− 1^ CaCl_2_, and 10 mmol L^− 1^ MES (pH 6.15)); Mock: treated for 2 h with the same volume of ethanol as the control after treatment with stomatal opening solution for 2 h; ABA: treated for 2 h with 20 μM ABA after treatment with stomatal opening solution for 2 h. Data were presented as the mean ± SD (*n* = 60) (^**^P < 0.01, Student’s t test)

Previous research indicates that ABA is an important inducer of stomatal closure, preventing water loss and thereby contributing to drought tolerance [18, 37]. We thus compared the stomatal aperture sizes of transgenic and control plants following ABA treatment. Scanning electron microscopy revealed that the percentage of closed stomata was almost two-fold higher in the transgenic plants than in the control plants (Fig. 5a, c). There were no significant differences in the average number of stomata between the control and transgenic plants. Thus, the observed reduced water loss rate and increased water use efficiency of the transgenic plants compared to the control plants were likely not due to differences in the number of stomata but to variability in stomatal closure (Fig. 5c).

To assess whether ABA induced stomatal closure differently in the transgenic plants, we compared the effects of exogenous ABA addition with that of mock. The average width of transgenic plant stomatal apertures was significantly smaller than that of the control plants for untreated (CK), mock-treated, or 20-μM ABA-treated plants. However, the width of stomatal apertures was more severely reduced for plants subjected to the 20-μM ABA treatment (Fig. 5e, Additional file 7). Thus, the transgenic plants heterologously expressing *rty* exhibited increased ABA-induced stomatal closure.

Trichomes affect the optical properties of the leaf surface and may protect plants from stress damage and reduced water loss through decreasing the rate of transpiration [38–40]. In the current study, we revealed that the density and number of epidermal trichomes on the abaxial side of leaves were higher in the transgenic plants than in the control plants. The abaxial surface per unit area of transgenic and control leaves were an average of 50 and 30 epidermal trichomes per unit area, respectively (Fig. 5b, d). The greater abundance of epidermal trichomes on the transgenic leaves may have aided in the increase in drought tolerance through reducing water loss and decreasing the rate of transpiration. These results suggest that the increase in drought tolerance is due to an increased number of epidermal trichomes and increased endogenous ABA concentrations, leading to smaller stomates.

### Expression of auxin biosynthetic and signaling genes is upregulated in transgenic plants

**Fig. 6:**
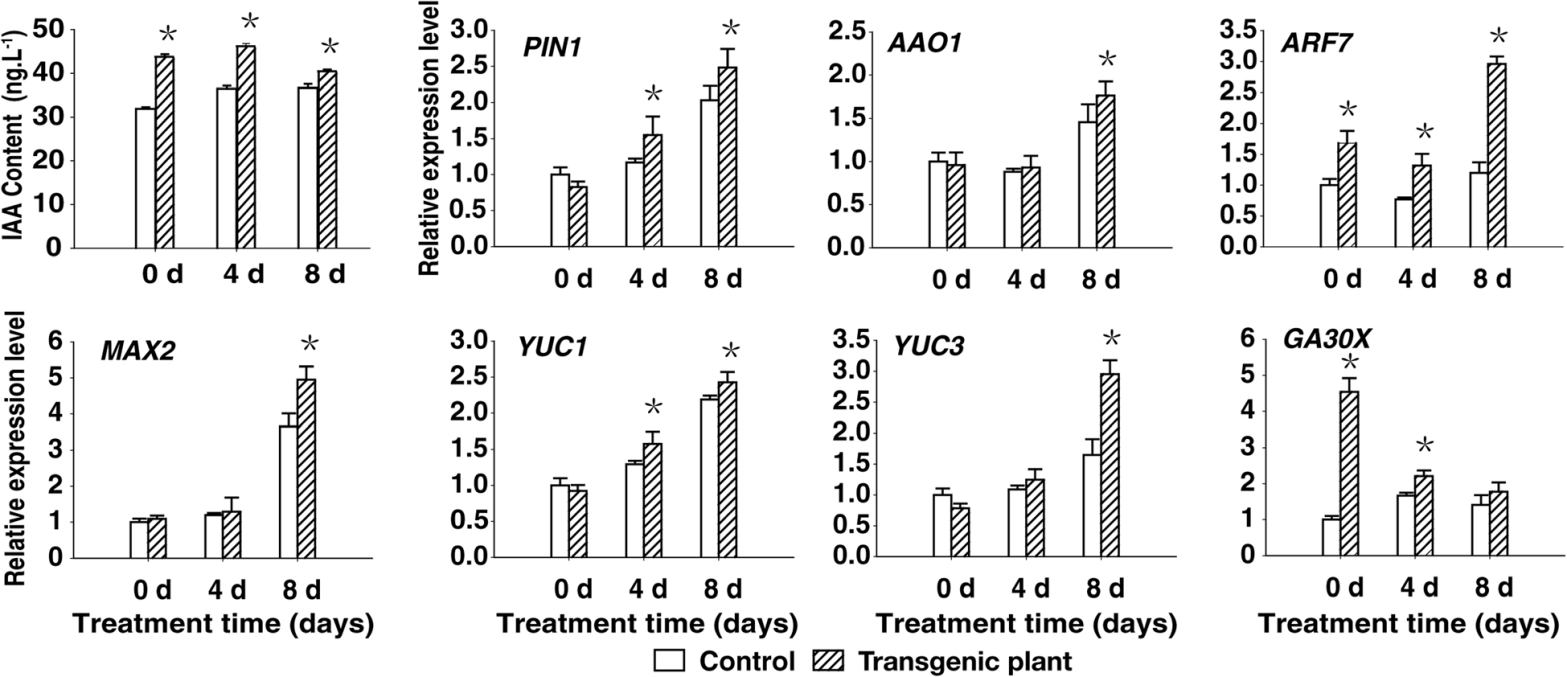
Accumulation of IAA and transcript levels of auxin-related genes. Transcript levels were quantified by an RT-qPCR assay, and *Actin* was used as a control. Two-month-old plants were subjected to drought stress by withholding water for 14 days. Leaves from 0 to 8 days were sampled every 2 days during the drought stress treatment. Seventy-eight pots control and transgenic plants were performed treatment, respectively. Data were presented as the mean ± SD (n = 3) (^*^P < 0.05, Student’s t test)

To determine the molecular mechanisms underlying the phenotypic differences between the control and transgenic plants, we compared the IAA contents of the control and transgenic plants during drought treatment. The concentrations of IAA in the control plants were 31.8 ng g^− 1^ on day 0, 36.5 ng g^− 1^ on day 4, and 36.7 ng g^− 1^ on day 8, but in transgenic plants were 43.0, 46.2, and 40.1 ng g^− 1^, respectively. The IAA content was always higher in the transgenic plants than in the control plants during drought treatment (Fig. 6). However, these results indicated that the IAA content in the transgenic and control plants did not increase during drought treatment.

To clarify the mechanism underlying the IAA content difference between the control and transgenic plants, we examined the expression levels of IAA biosynthetic and signaling genes, including *PIN1*, *AAO1*, *ARF7*, *MIX2*, *YUC1*, *YUC3*, and *GA3ox*. RT-qPCR analysis indicated that drought treatment upregulated the expression of the IAA biosynthetic and signaling genes in the transgenic plants.

These results suggest that the heterologous expression of *rty* in transgenic plants induced the expression of IAA biosynthetic and signaling genes, which in turn increased IAA accumulation. Furthermore, the increased ABA levels during drought treatment (Fig. 6) likely influence the observed production of additional roots (Fig. 2b, c) and trichomes (Fig. 5b, d) in the transgenic plants.

### Expression of stress-inducible genes and ABA biosynthetic genes is upregulated in the transgenic plants

**Fig. 7:**
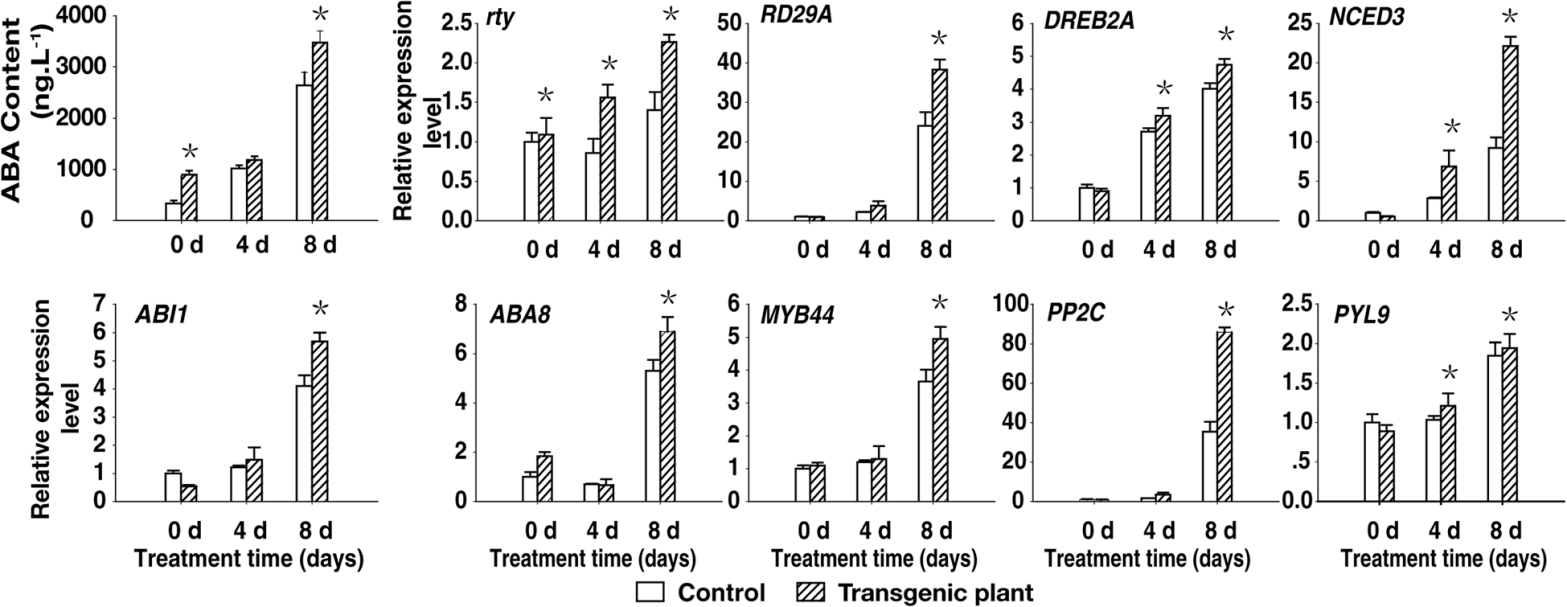
Accumulation of ABA and expression of ABA-related genes. Transcript levels were quantified by an RT-qPCR assay, and *Actin* was used as the control. Two-month-old plants were subjected to drought stress by withholding water for 14 days. Leaves from 0 to 8 days were sampled every 2 days during the drought stress treatment. Data were presented as the mean ± SD (n = 3) (^*^P < 0.05, Student’s t test)

Under drought conditions, ABA concentrations increase to a specific threshold by midday, inducing ion efflux and inhibiting sugar uptake by guard cells, after which the stomatal apertures decrease in size for the rest of the day [41]. To elucidate the role of ABA during stress responses, we compared the ABA contents of the two-month-old control and transgenic plants following a drought treatment. The concentrations of ABA in the control were 334.0 ng g^− 1^ on day 0, 1017.2 ng g^− 1^ on day 4, and 2635.3 ng g^− 1^ on day 8, but in transgenic plants were 939.3, 1083.7, and 3471.3 ng g^− 1^, respectively. The ABA concentration was significantly higher at 8 days after initiating the drought treatment in both the control and transgenic plants, but was 1.3-fold higher in the transgenic plants (Fig. 7).

To determine the molecular mechanisms underlying this difference in ABA contents between the control and transgenic plants, we examined the expression of genes involved in ABA biosynthesis, catabolism, transport, and signaling, as well as drought-responsive genes, including the following: *NCED3* [32], *ABI1* [33], *RD29A* [34], *DREB2A* [35], and *PP2C* [36]. Water deficit stress promotes ABA biosynthesis via the upregulated expression of *NCED3* [42]. RT-qPCR analysis indicated that the *NCED3* transcript levels were significantly higher at 8 days after starting the drought treatment, with the increase being more pronounced in transgenic plants, implying that this gene was actively expressed. Additionally, the expression levels of the ABA-inducible marker genes (*RD29A* and *DREB2A*), ABA biosynthetic genes (*ABI1*, *ABA8*, and *PYL9*), a stomatal closure-responsive gene (*PP2C*), and *MYB44* were higher in the transgenic plants than in the control plants during the drought treatment (Fig. 7). High *PP2C* and *MYB44* transcript levels may induce stomatal closure [36].

Thus, the heterologous expression of *rty* in strawberry plants considerably increased ABA accumulation. The expression of stress-inducible genes and ABA biosynthetic genes may then trigger stomatal closure via an ABA-dependent pathway, which may contribute to the observed drought tolerance of the transgenic plants.

## Discussion

**Fig. 8:**
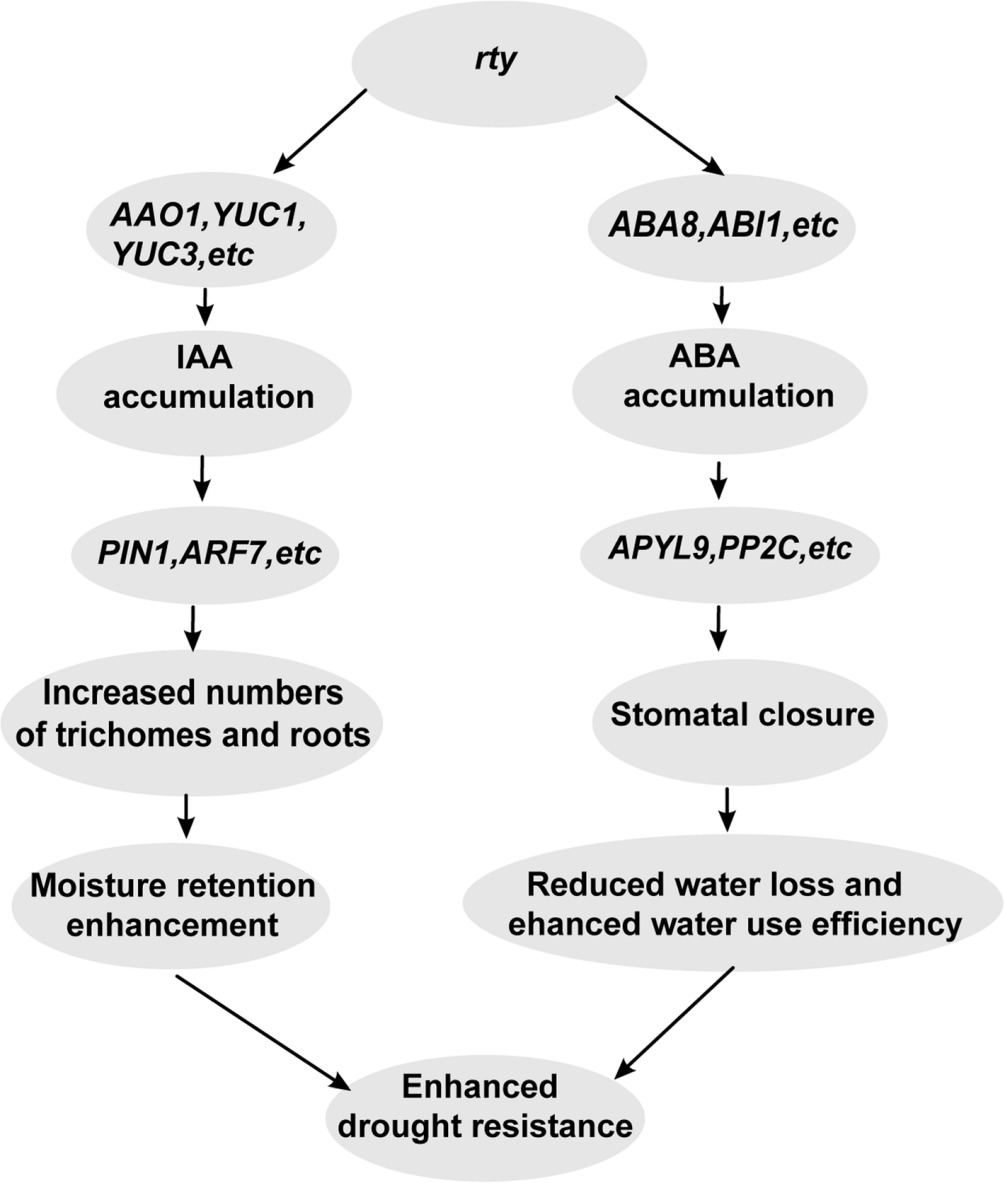
Molecular mechanism underlying *rty* gene improving hormone-mediated drought tolerance in plant. In plants, *rty* promotes IAA and ABA accumulation. The transcript levels of IAA biosynthetic genes, including *AAO1*, *YUC1*, and *YUC3*, increase auxin accumulation. The auxin induces transcripts of related response genes including *PIN1* and *ARF7*, and the products of excess roots and increases the density of leaf trichomes, which keep moisture retention. The transcript levels of ABA biosynthetic genes including *ABI1* and *ABA8*, improve ABA accumulation. The ABA induces transcripts of related response genes including *PYL9* and *PP2C*, and promote stomatal closure in the plants, which increase the water use efficiency and decreased the water loss rate. Plant photosynthesis, respiration, and transpiration are all affected by the *rty* gene. These changes contributed to the increased tolerance of the plants to drought stress

The *rty* gene improves hormone-mediated drought tolerance in plants (Fig. 8). In plants, *rty* promotes IAA and ABA accumulation [12, 14, 15]. The transcript levels of IAA biosynthetic genes, including *AAO1*, *YUC1*, and *YUC3*, increase auxin accumulation [43]. The auxin induces transcripts of related response genes including *PIN1* and *ARF7*, and the products of excess roots and increases the density of leaf trichomes, which keep moisture retention [44, 45]. The transcript levels of ABA biosynthetic genes including *ABI1* and *ABA8*, improve ABA accumulation [33, 46]. The ABA induces transcripts of related response genes including *PYL9* and *PP2C*, and promotes stomatal closure in the plants, which increase the water use efficiency and decreased the water loss rate [47, 48]. Plant photosynthesis, respiration, and transpiration are all affected by the *rty* gene. These changes contributed to the increased tolerance of the plants to drought stress [49–53]. In this study, we demonstrated that the heterologous expression of rty in strawberry improved drought tolerance by promoting auxin and ABA accumulation. These phytohormones together brought about various physiological changes that improved drought tolerance, such as increased root production, trichome density, and stomatal closure.

### Heterologous expression of *rty* increases the drought tolerance of transgenic strawberry plants

Transgenic strawberry plants heterologously expressing *rty* exhibited a strong growth potential (Fig. 1d), and produced more roots (Fig. 2b, c) and leaf trichomes (Fig. 5b, d), but fewer tillers (Fig. 1g), than the control plants. Measurement of IAA concentrations revealed that the heterologous expression of *rty* significantly increased the IAA levels in transgenic strawberry plants (Fig. 1h). The high IAA contents of the transgenic strawberry plants resulted in earlier root development and increased numbers of roots. These dominant effects were consistent with the general functions of *rty* in *A. thaliana*. Moreover, *rty* expression appears to be critical for regulating IAA concentrations in *A. thaliana*. A recessive *rty* mutation also yields high endogenous IAA concentrations. The most extreme phenotypic effects of *rty* expression are the proliferation of adventitious roots, lateral roots, and the restriction of shoot development. These phenotypes are most likely caused by increases in auxin concentrations [6–8, 10, 12]. Thus, *rty* plays a critical role in regulating endogenous auxin concentrations to facilitate normal growth and development.

In the current study, we revealed a hitherto unknown function of *rty* related to drought tolerance, revealing that heterologous expression of this mutant gene increases ABA concentrations in response to drought stress. Thus, *rty* helps regulate plant growth and development as well as responses to abiotic stress conditions. Specifically, the heterologous expression of *rty* significantly increased the ABA content of the transgenic plants (Fig. 1i). The transgenic plants had a lower water loss rate, exhibited less electrolyte leakage, and had higher water use efficiency than the untransformed controls (Fig. 4). The observed accumulation of ABA in the transgenic plants in response to drought treatment suggested that the increased drought tolerance of the transgenic plants was mediated by an ABA-dependent pathway.

Previous reports have indicated that ABA is important for stomatal closure, which limits water loss and enhances drought tolerance [18, 54]. Because ABA helps regulate stomatal closure [55], we speculate that *rty* expression maintains narrower stomatal apertures in plants exposed to drought stress, which reduces water loss through transpiration. We determined that the heterologous expression of *rty* in strawberry likely increases the sensitivity of the transgenic plants to ABA and improves their tolerance to drought stress.

### *rty* expression promotes drought stress responses via ABA-regulated stomatal closure

Plant drought responses involve a complex process regulated by multiple molecular and cellular pathways. The expression levels of some genes are upregulated or downregulated by exposure to abiotic stresses, and the heterologous expression of these genes can increase the tolerance of transgenic plants to drought or salt stress [56–59]. Consistent with this, our results indicated that the eheterologous expression of *rty* has critical effects on strawberry drought responses. Specifically, heterologous expression of *rty* increased the tolerance of the transgenic strawberry plants to drought stress. Moreover, the transgenic plants produced more leaf trichomes and had a higher percentage of closed stomata and smaller stomatal apertures compared to the control plants (Fig. 5, Additional file 7). When plants are subjected to drought stress, some physiological factors (e.g., electrolyte leakage and POD and CAT activities) may be quickly activated to enable these plants to survive under extreme environmental conditions [60–63]. Thus, physiological indices related to plant osmotic stress caused by drought may be useful measures for quickly and accurately assessing plant resistance to abiotic stress. Electrolyte leakage, which reflects the degree of cell membrane damage [60], was higher in the leaves of control plants than in those of the transgenic plants (Fig. 4). The results suggested that heterologous expression of *rty* may strengthen the plant cell membrane integrity in response to drought stress. Furthermore, POD and CAT are vital antioxidant enzymes that protect plants from abiotic stress damage [64, 65]. In this study, we established that CAT and POD were more active in the transgenic plants than in the control plants (Fig. 3d, e). This information may be useful for clarifying the mechanism underlying the increased drought tolerance of the transgenic plants.

In *A. thaliana*, many positive and negative regulators have been identified and characterized as key components of ABA biosynthesis and drought signaling [18, 66]. Biotic stresses upregulate the expression of several ABA biosynthetic genes [67–72]. Water stress-induced ABA accumulation is preceded by significant increases in *Phaseolus vulgaris CED1* transcript and protein levels in the leaves and roots [42, 73, 74]. In *A. thaliana*, of the five *NCED* genes involved in ABA biosynthesis, the expression of only *AtNCED3* is strongly induced by dehydration, although a minor increase in the expression of the other *NCED* genes has also been reported [42, 74, 75]. Additionally, *AtNCED3* overexpression in transgenic *A. thaliana* plants increased the ABA content and desiccation tolerance [42]. Similarly, in the current study, some ABA biosynthetic and abiotic stress-responsive genes, including *RD29A*, *DREB2A*, *NCED3*, *ABI1*, *ABA8*, *PYL9*, and *PP2C* [67–71, 76–78], were more highly expressed in the transgenic plants than in the control plants under drought conditions (Figs. 6, 7). These results imply that the increased tolerance of the transgenic plants to drought stress may be influenced by the upregulated expression of these genes in response to drought conditions. Further study will focus on the how *rty* interacts with these ABA biosynthetic pathways and with stress-induced signal transduction during the plant’s response to drought stress. In addition to providing a foundation for future studies of drought stress tolerance, the results presented here may be used to develop genetically modified strawberry varieties with improved drought tolerance.

## Conclusion

In this study, *rty* gene was isolated from *Arabidopsis thaliana* and placed under the control of the cauliflower mosaic virus (CaMV) 35S promoter in the pBI121-rty binary vector carrying the selectable marker of neomycin phosphotransferase II (*NPT* II). Transgenic strawberry plants were confirmed by PCR and western blot analysis. The transgenic strawberry plants induced IAA accumulation and increased the production of adventitious roots as well as trichomes on the abaxial leaf surface. Also, the ABA accumulation increased stomatal closure in the transgenic strawberry plants. This enhanced water use efficiency, reduced water loss rate, and improved more drought tolerant. A novel function was revealed for *rty* related to ABA and drought responses. Transgenic approaches can be used to overcome the inherent trade-off between plant growth and drought tolerance by enhancing water use efficiency and reducing water loss rate under water shortage conditions. This study provides the basis for future genetic modifications of strawberry to improve drought tolerance.

## Methods

### Vector construction for heterologous expression of *rty*

A previous study [8] and BLAST analysis (GenBank accession: AY050987) suggested that *rty* encodes an aminotransferase or a C-S lyase that catalyzes IAA biosynthesis. To functionally characterize *rty* in strawberry (*Fragaria* × *ananassa*), we created an overexpression construct by PCR-based cloning. A cDNA fragment from an *A. thaliana* mutant (Stock Number CS8156 from Arabidopsis Biological Resource Center) containing the entire *rty* coding region was amplified as previously described [69]. The PCR products and the pBI121 vector were digested with *Sma* I and *Sac* I endonucleases, and the digested PCR and vector products were then ligated with T4 DNA ligase (Promega Corporation, Madison, USA). *rty* gene was isolated from *Arabidopsis thaliana* and placed under the control of the cauliflower mosaic virus (CaMV) 35S promoter in the pBI121-rty binary vector carrying the selectable marker of neomycin phosphotransferase II.

### Heterologous expression of *rty* in strawberry plants

A strawberry cultivar ‘Honeoye’ (*Fragaria × ananassa* Duch.) were used as the experiment materials. The strawberry cultivar ‘Honeoye’ was developed at Cornell University by plant breeders at the New York State Agricultural Experiment Station and released in 1979. ‘Honeoye’ was introduced to China as commercial cultivar in 1983. The pBI121-*rty* binary vector was transformed into *Agrobacterium tumefaciens* GV3101 using a freeze-thaw procedure [79]. *Agrobacterium-mediated* transformation of ‘Honeoye’ strawberry was carried out according to the protocol of Jin and Wang [80]. The leaf disc method was used to transform strawberry. The leaves of sterile seedlings subcultured for 25–30 days were cut into 3–5 mm^2^ leaf discs. *Agrobacterium tumefaciens* LBA4404 carrying the target gene was cultured in liquid LB medium containing 100 mg L^− 1^ kanamycin at 28 °C for 16 h. The leaf discs were placed in this culture for 5 min, transferred to regeneration MS medium (Murashige and Skoog, 1962) containing 6-BA 3.0 mg L^− 1^ and 2, 4 - D 0.1 mg L^− 1^, cultured in darkness for 1 d; and then transferred to regeneration medium containing 400 mg L^− 1^ cephalosporin and 5 mg L^− 1^ kanamycin, at 25 ± 1 °C, a light cycle of 16 h, and a light intensity of 30 μmol m^− 1^ s^− 1^. After 30 days of culture on subculture medium (MS + 6-BA 0.2 mg L^− 1^ + IBA 0.1 mg L^− 1^), the shoots of 3 ~ 5 cm in height had formed and these were rooted on root medium (1/2 MS + IBA 0.2 mg L^− 1^). Kanamycin-resistant plants were further confirmed by a PCR-based assay using *NPTII* and *rty* genespecific primers (Table 1). Western blot analysis was used to verify that the target protein was produced in the transgenic plants, which were then used for subsequent analyses [86]. Untransformed strawberry was used as the control for analysis of drought stress.

**Table 1:** The sequences of the primers used in this work for vector construct, screening the positive transform, and transcript assay

Gene	Accession	Primer Sequence (5′ → 3′) ^a^	Protein	Ortholog gene in *Arabidopsis*	Function	Reference (s)
*rty*	AY050987	F: AAGCTTATGAGC GAAGAACAACCACACGC R: GAGCTCTTACAT TTCGAGATTATTATCACTC	tyrosine aminotransferase	AT2G20610	Regulation of cell growth by extracellular stimulus, glucosinolate biosynthetic process, adventitious root development, indoleacetic acid biosynthetic process	[6]
*NPTII*	AAL92039	F: CCGGTATAAAGGGACCACCT R: ATGTTGCTGTCTCCCAGGTC	neomycin phosphotransferase II	–	Neomycin phosphotransferase II as selectable markers in transformed plant screening	[81]
*Actin*	XM_004306544	F: GGCCGTTCTCTCTCTGTATGC R: TTCTGGGCACCTGAATCTC	Actin	–	Actin	[29]
*FvRD29A*	XM_011472069	F: AGAGGCACGCCACGAATA R: GCCAGTTTGGTCCTTGCT	Cold regulated protein	AT5G52310	Responded to abscisic acid, cold, desiccation, mannitol, osmotic stress, reactive oxygen species, salt stress, water deprivation, and wounding	[34]
*FvNCED3*	XM_004300619	F: TCACCACAAGAGATTCCTTTCT R: ATGGCTGTGAGTAGGTTGGA	Encodes 9-cis-epoxycarotenoid dioxygenase	AT3G14440	Regulated in response to drought and salinity	[32]
*FvDREB2A*	XM_004307642	F: TGCTGCGAGTCTACTACGATGTCT R: TGATCCATAGGAACCTCGCTTT	Encodes a transcription factor binds to DRE/CRT cis elements	AT5G05410	Regulates expression of many water stress–inducible genes.	[35]
*FvABA8*	XM_004300635	F: ATGGAACTACTCAGCCTCAACT R: ATGGCGAGCGGAAATGAG	Encodes a protein with ABA 8′-hydroxylase	AT4G19230	Involved in ABA catabolism.	[46]
*FvABI1*	XM_011469096	F: GCATGGCTAAAGCTAGTAACAGAC R: GCTCCTCCAGTGTTATCTTCTTGT	Protein phosphatase 2C family protein	AT4G26080	Involved in abscisic acid (ABA) signal transduction	[33]
*FvPYL9*	XM_004306638	F: GGGAGTCTTAGGGAAGTGAATG R: GGATGGACGGTGATAATAGAAGAG	Encodes regulatory components of ABA receptor	AT1G01360	Interacts with and regulates the type 2C protein phosphatases (PP2Cs).	[78]
*FvPP2C*	XM_004291463	F: ACGAAATATGCCGAGAAGCGAAAG R: TTGGCGGAAATCGTTAGGCTCAT	Encodes a member of the group A protein phosphatase 2C	At1g07430	Negative regulation of abscisic acid-activated signaling pathway.	[36]
*FvAAO1*	XM_004296223	F: TCAACCGCTGCTCCATCACG R: GTCGGTCAGTGGTGTTTTGGGC	Encodes aldehyde oxidase 1	AT5G20960	Involved abscisic acid biosynthetic process, auxin biosynthetic process.	[76]
*FvPIN1*	XM_004303589	F: CAAAGCCGCAAAGATAGAGCC R: TGGGATGCCCTGACCTGAT	Encodes an auxin efflux carrier involved in shoot and root development	AT1G73590	Involved in the maintenance of embryonic auxin gradients	[82]
*FvARF7*	XM_004287612	F:ACAATCACTGGCATTAGCGAGC R: GAGGTGGGCAGATGTAGAAAGG	Encodes an auxin-regulated transcriptional activator	AT5G20730	Involved in auxin-activated signaling pathway, lateral root development, lateral root formation.	[83]
*FvMAX2*	XM_011468656	F:ACTGTGGCGATTTGACGGAT R: CCACAACAAAGAAGCCAGCGT	F-box leucine-rich repeat family of proteins	AT2G42620	Responses to abiotic stress conditions.	[84]
*FvYUC1*	JF898837	F: CAAATGGTTGGAAAGGTGAGC R: CGAGGACATTGAGCGGTGTTGGA	Encodes a member of the YUC family	AT4G32540	Involved in auxin biosynthetic process.	[72]
*FvYUC3*	JX417080	F: ATTTCCCAACCTACCCAACC R: AGTCTTGACGAGCCAAAGTC	Encodes a member of the YUC family	AT1G04610	Involved in auxin biosynthetic process, oxidation-reduction process, response to ethylene.	[72]
*FvGA3ox*	XM_004302902	F: CCTGTAAGAATCTCCGAAGCC R: CGTGTAGTGAGTTGAAGTCTGC	gibberellin 3-beta-dioxygenase activity,	AT1G15550	Involved in later steps of the gibberellic acid biosynthetic pathway.	[85]

^a^*F* forward primer, *R* reverse primer. Underlined characters indicate the restriction enzyme cutting site

The root and leaf tissues of the control and transgenic plants cultured on subculture medium MS + 6-BA 0.2 mg L^− 1^ + IBA 0.1 mg L^− 1^ were placed in liquid nitrogen and ground in a mortar. Immediately after evaporation of the liquid nitrogen, 2 mL of extraction buffer containing 50 mmol L^− 1^ Tris, 50 mmol L^− 1^ EDTA, 100 mmol L^− 1^ KCL, 2 mmol L^− 1^ dithiothreitol (DTT), 2 mmol L^− 1^ PMSF, and 10% (w/v) glycerine (pH 7.5), was added. Samples were extracted for 2 h at 0 °C and then centrifuged at 12,000×g for 15 min at 4 °C. The concentration of protein in the supernatant was determined using the Bradford assay. All samples were stored at − 70 °C. After SDS-PAGE separation, the resolved proteins were electroblotted to a PVDF membrane. Electroblotted membranes were subjected to western blot analysis using anti-rty serum (Beijing Protein Innovation Co., Ltd., China). To increase the serum specificity, the Atrty protein secondary structure, tertiary structure, hydrophobicity, antigenicity, and specificity were analyzed. Recombinantly expressed rty protein (1–131 aa) was used as the immunogen to generate antibody (Additional file 1). Membranes were then treated with alkaline phosphatase-labelled protein and goat anti-rabbit antibody (Jinqiao, Co., Ltd., Beijing, China), and then dyed with BeyoECL Plus kit (Beyotime Biotechnology Co., Ltd., Shanghai, China).

### Quantitative analysis of indole-3-acetic acid (IAA) and abscisic acid (ABA)

Three independent transgenic lines 3, 6, and 8 were examined for all physiology and molecular assays (Fig. 1e). The data from transgenic plants in this study are the average of these three independent lines. Indole-3-acetic acid (IAA) and abscisic acid (ABA) content in tissues of the control and transgenic plants were assessed by high-performance liquid chromatography-mass spectrometry according to the method of Pan et al. [87]. One-month-old control and transgenic plants were inoculated on subculture medium for 4 days with no exogenous growth regulators. The first day after inoculating on subculture medium was defined as day 0. Basic shoot segments (~ 0.4 cm) of control and transgenic plants were sampled on days 0 and 4. Around 50 mg of fresh samples was frozen in liquid nitrogen, ground to powder, and transferred to 2-mL screw-cap tubes. Metabolites were immediately extracted from the powdered tissue by adding 500 μl extraction solvent (2-propanol: H_2_O: concentrated HCl, 2:1:0.002, v/v/v), to each tube on a shaker at a speed of 100 rpm for 30 min at 4 °C after the addition of stable isotopes, which were used for isotope dilution-based quantification. Samples were re-extracted by adding 1 mL dichloromethane and spun at a speed of 100 rpm for 30 min at 4 °C. Samples were then centrifuged at 4 °C in an Avanti J-26XP centrifuge at 13,000×g for 5 min (Beckman Coulter, USA). Two phases formed during centrifugation, with a layer of plant debris between the layers. An aliquot of ~ 900 μL from the lower phase was transferred to a screw-cap vial using a Pasteur pipette and concentrated using a nitrogen evaporator. Solids were redissolved in 0.1 mL methanol and centrifuged at 13,000×g for 5 min at 4 °C. An aliquot of 50 μl of the resulting supernatant was injected into an Agilent 1260 Infinity series HPLC system (Agilent Technologies, Santa Clara, CA) for chromatographic separation before detection by tandem mass spectrometry (MS/MS) using an AB SCIEX QTRAP 5500 LC/MS/MS System (AB SCIEX Deutschland GmbH, Darmstadt, Germany).

### Histological observations

Histological observations of root development were conducted. One-month-old control and transgenic plants were cultured on subculture medium (MS + 6-BA 0.2 mg L^− 1^ + IBA 0.1 mg L^− 1^) for 0–9 days. About 0.2 cm basic shoot segments of control and transgenic plants were sampled every day. The samples were fixed in formaldehyde-acetic acid fixative solution (50% ethanol-formalin-acetic acid =18:1:1) for 48 h. The next method was performed as previously described [88].

### Drought treatment

The transgenic and control plants were subjected to a drought tolerance test. Pots (16 × 16 cm) with the same volume of nutrient soil (peat soil: field soil: vermiculite at 1: 1: 1) per pot. After saturation with 0.8 L of water, the 30-day-old control and transgenic plants cultured on root medium were planted in pots (one plant per pot). Two month later, these plants were subjected to drought stress by withholding water for 14 days after saturation with 0.8 L of water. About seventy-eight pots of control and transgenic plants were analyzed per treatment, respectively. The pot positions were often changed to minimize the effects of environmental variability in the greenhouse. Leaves were sampled every 2 days during the drought stress treatment. Three pots were sampled. For the drought treatment, the relative water content was measured every 2 days using a Soil Temperature/Moisture Meter L99-TWS-1(Shanghai Danding International Trade Co., Ltd). Before the drought treatment, the mixed vermiculite and soil was saturated with 0.8 L water. The soil water content before drought was set to 100%, while the relative soil water content after the 14-day drought treatment was ∼6%. Additionally, plants were rewatered after the 14-day drought treatment and the survival rate was calculated 7 days later.

### Simulated drought stress with PEG 8000

Polyethylene glycol (PEG)-infused medium was prepared as described by Verslues et al [89]. Because PEG cannot be dissolved in agar before pouring medium, PEG-infused medium was prepared by pouring a liquid medium containing PEG on top of the solidified agar. An aliquot of 5 mmol L^− 1^ MES was added to stabilize the pH of the medium (to pH 5.7) and to avoid having to adjust the pH after adding PEG. An appropriate volume of ½ MS containing 15 g L^− 1^ agar and 5 mmol L^− 1^ MES was prepared for the agar medium. While the agar medium was still hot, an equal volume of agar medium was divided into the glass flasks, autoclaved, and then frozen. An appropriate volume of ½ MS liquid medium containing 5 mmol L^− 1^ MES (to pH 5.7) was then prepared. Solid PEG 8000 (Sigma Catalog number P-2139) was weighed out at 10, 20, 30% (w/v) into the liquid medium after autoclaving, while it was still hot. The PEG- infused liquid medium was filter sterilized with a 0.45 μm filter. The 0.04 L volume of PEG- liquid medium was pipetted on top of the glass flasks containing solidified agar. The 0.06 L volume of liquid medium was pipetted on top of the glass flasks containing solidified agar, as the 0 treatment. The agar was added to the PEG liquid medium at a ratio of 2:3. The glass flasks were allowed to equilibrate for 24 h at room temperature. Before use, the PEG liquid medium was poured off, careful not to dislodge the agar which may no longer be tightly adhered to the bottom of the glass flasks. Thirty-day-old control and transgenic plants heterologously expressing *rty* on subculture medium were transferred to glass flasks containing 0% (CK), 10, 20, and 30% PEG-infused medium at 21 °C in a temperature-controlled growth room with a 16-h light/8-h dark photoperiod. Forty-eight hours later, the treated plants were observed, photographed, sampled, and subjected to RT-qPCR and antioxidant enzyme activity analyses.

### Gas exchange measurements and water loss

Photosynthetic parameters were measured as described by Zhao et al. [31]. Three independent the control and transgenic plants were analyzed and the experiment was replicated. Gas exchange measurements were taken with the LI-6400 (LI-6400, Li-Cor, USA). Every three mature and fully expanded leaves from three transgenic plants and wild type with good growth status and proper position were randomly sampled. The measurements were taken on two hourly basis from 8:00 to 16:00 in the clear day of April 24–25, 2017. Water Use Efficiency (WUE) was calculated the ratio net photosynthetic rate divided by transpiration rate [90]. To assess the water loss, leaves of one-month-old plants from the same position were weighed with an electronic scale, and then placed in a glass culture dish on a layer of filter paper and weighed every hour.

### Measurement of antioxidant enzyme activities

To measure antioxidant enzyme activities, fresh leaves (0.2 g) from the control and transgenic plants were cut into small pieces and ground with a mortar and pestle on ice, in a solution comprising 1 mL 0.05 M phosphate buffer (pH 7.8), 3 g polyvinylpyrrolidone, and 0.1 g quartz sand. The mortar was washed twice with 2 mL phosphate buffer (pH 7.8). The resulting solution was poured into a 10-mL centrifuge tube for a final volume of 7 mL with phosphate buffer (pH 7.8). The tube was centrifuged at 2500×g for 20 min at 4 °C. The supernatant was collected, topped up to 10 mL with phosphate buffer (pH 7.8), and used as the enzyme solution for the subsequent analysis of antioxidant enzyme activities. Specifically, catalase (CAT) activity was assayed at 240 nm. The sample was assayed again 1 min later. The peroxidase (POD) activity in a 0.5-mL aliquot of the enzyme solution was determined at 470 nm. The sample was assayed again 1 min later. The CAT and POD activities were calculated as described by Zhao et al [31].

### Analysis of stomatal closure and plant trichomes by scanning electron microscopy

A hole puncher (0.5 cm diameter) was used to collect leaf discs from two-month-old control and transgenic plants at 8 h, 10 h, 12 h, 14 h, 16 h, and 18 h. The collected samples were immediately fixed for 24 h in fixing solution (90 mL of 50% [v/v] ethanol, 5 mL glacial acetic acid, 5 mL formaldehyde) (using a vacuum pump to remove the gas attached to the tissue surface). The leaf discs were dehydrated with 70, 80, 90, 95, and 100% ethanol for 15–20 min, respectively, and then dried with CO_2_ and coated on film. The stomatal and plant trichomes were observed and photographed with a Hitachi S-4800 scanning electron microscopy system. Three biological replicates were performed.

For ABA-induced stomatal closure, leaf discs were obtained with a hole puncher (0.5 cm diameter) from four different two-month-old plants at 12:00 and were then incubated in stomatal opening solution (50 mmol L^− 1^ KCl, 10 mmol L^− 1^ CaCl_2_, and 10 mmol L^− 1^ MES (pH 6.15)) for 2 h in a growth chamber with a light intensity of 130 mmol m^− 2^ s^− 1^ at 22 °C. Aliquots of 20 μmol L^− 1^ ABA (dissolved in absolute ethanol) or mock (the same volume of absolute ethanol) were added to the opening solution, and the leaf discs were incubated for an additional 2 h. Then, all samples were immediately fixed, dehydrated, dried, and coated. Three images of CK (not treated with stomatal opening solution and ABA), ABA-treated, or mock-treated leaf discs were obtained using scanning electron microscopy. The stomatal apertures (widths and lengths) of 60 stomata were measured per leaf disc. The mean stomatal apertures were determined based on measurements from four leaf discs.

### Measurement of electrolyte leakage

To examine electrolyte leakage, fresh leaves (0.2 g) from control and transgenic plants (one-month-old plants cultured on subculture medium) were cut into smaller pieces and placed in a glass tube containing 10 mL deionized water. The samples were mixed on a gyratory shaker (at about 150 rpm) for 6 h at room temperature. After the initial conductivity (Ci) was measured with a DDS-307A conductivity meter (Leici), the samples were boiled for 20 min to release the electrolytes into the solution. After the samples were cooled to room temperature, the conductivity of the dead tissue (Cmax) was measured. Relative electrolyte leakage was calculated as (Ci/Cmax) × 100%.

### Reverse transcription quantitative PCR analysis

The expression levels of specific genes in the control and transgenic plants were analyzed using a reverse transcription quantitative PCR (RT-qPCR) assay. Total RNA and cDNA were prepared as previously described [91]. RT-qPCR primer sequences are provided in Table 1. Two independent biological replicates and three technical replicates were performed. The resulting data were analyzed according to the 2^−ΔΔCt^ method as outlined by Livak and Schmittgen [92]. The expression levels of specific genes were normalized against the *Actin* expression level.

## Supplementary Information

**Additional file 1.** Immunogen sequence analysis of rty protein. Rty protein secondary structure, tertiary structure, hydrophobicity, antigenicity, and specificity were analysis. Rty protein (1-131aa) recombinant protein expression was used as immunogen to keep away from the protein binding site.

**Additional file 2.** Transgenic plants were confirmed by PCR.

**Additional file 3.** Transgenic plants were further analyzed by western blot with anti-rty.

**Additional file 4.** Transgenic plants were further analyzed by western blot with anti-actin.

**Additional file 5 **Relative water content during 0–14 days under drought stress in the control and transgenic plants. Relative water content was measured using the soil temperature/moisture meter every 2d during 0–14 days of drought stress. Three biological replicated were performed. Since the mixed vermiculite and soil was saturated with 0.8 L water, the relative soil water content before drought was set as 100%. Data are presented as the mean ± SD (*n* = 3).

**Additional file 6 **Relative expression levels assay during the PEG treatment in the control and transgenic plants. Relative expression levels were quantified by a RT-qPCR assay, which used *Actin* as a control. The 30 days old control and transgenic plants heterologously expressing *rty* cultured for on MS medium adding 6 - BA 0.2 mg L^− 1^ and IBA 0.1 mg L^− 1^ were transferred to 100-mL glass flasks containing 0% (CK), 10, 20%, or 30% PEG-infused medium at 21 °C in a temperature-controlled growth room with a 16-h light/8-h dark photoperiod. Forty-eight hours later, the treated plants were sampled to assay. Three biological replicates were performed per treatment. Data are presented as the mean ± SD (n = 3) (^*^P < 0.05, Student’s t test).

**Additional file 7.** ABA-induced stomatal closure by scanning electron microscopy in the control and transgenic plants. (a) and (b) control and transgenic plant were no treated with stomatal opening solution and ABA; (c) and (d) control and transgenic plant were treated 2 h with the same volume of ethanol as a control after treatment with stomatal opening solution 2 h; (e) and (f) control and transgenic plant were treated with the 20 μM ABA 2 h after treatment with stomatal opening solution 2 h.

## Data Availability

All data generated or analyzed during this study are included in the published article and its supplementary data files (Figs. 1, 2, 3, 4, 5, 6, 7, and 8 and Additional files 1, 2, 3, 4, 5, 6, and 7). The datasets used and/or analyzed during the current study are available from the corresponding author Wanmei Jin.
