# Supplementary figures and images for: Heterologous expression of Arabidopsis thaliana rty gene in strawberry (Fragaria × ananassa Duch.) improves drought tolerance
Source: BMC Plant Biol. 2021 Jan 21;21:57. doi: 10.1186/s12870-021-02839-4 (PMC7818561; doi:10.1186/s12870-021-02839-4)

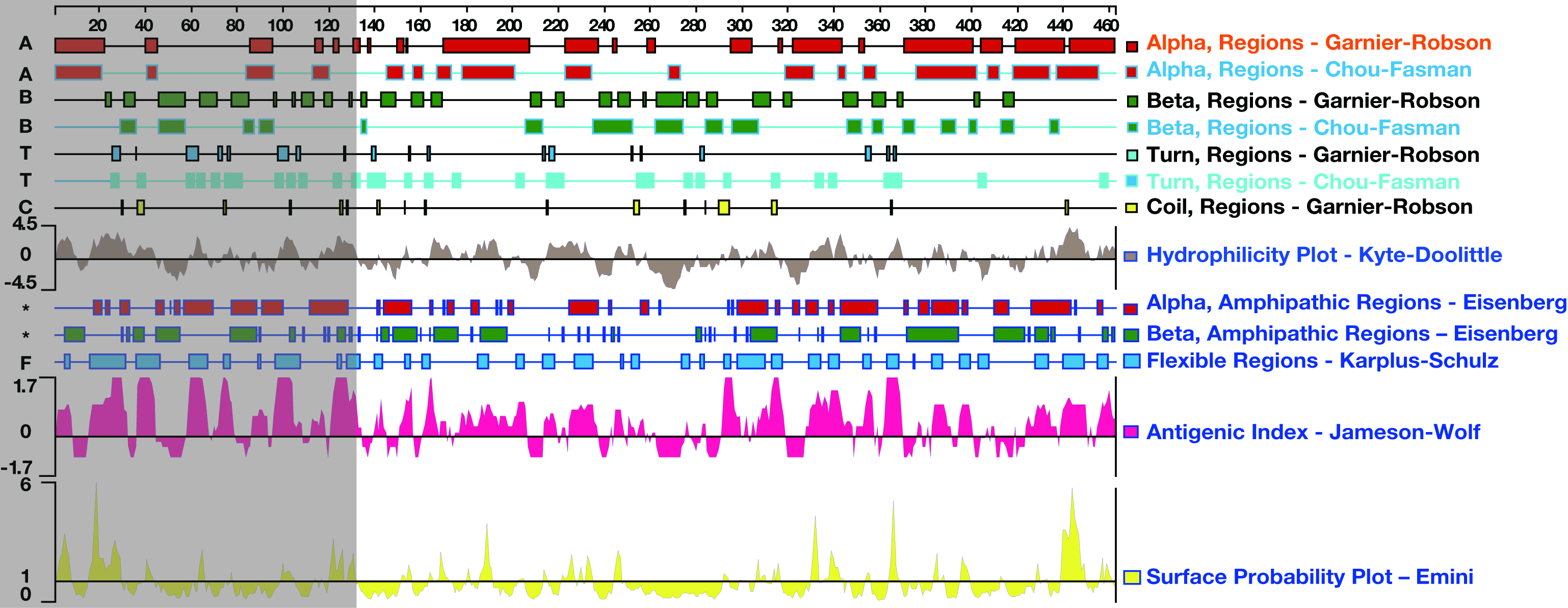

Supplement: Supplementary file 1 — Additional file 1. Immunogen sequence analysis of rty protein. Rty protein secondary structure, tertiary structure, hydrophobicity, antigenicity, and specificity were analysis. Rty protein (1-131aa) recombinant protein expression was used as immunogen to keep away from the protein binding site. [file 12870_2021_2839_MOESM1_ESM.tif]

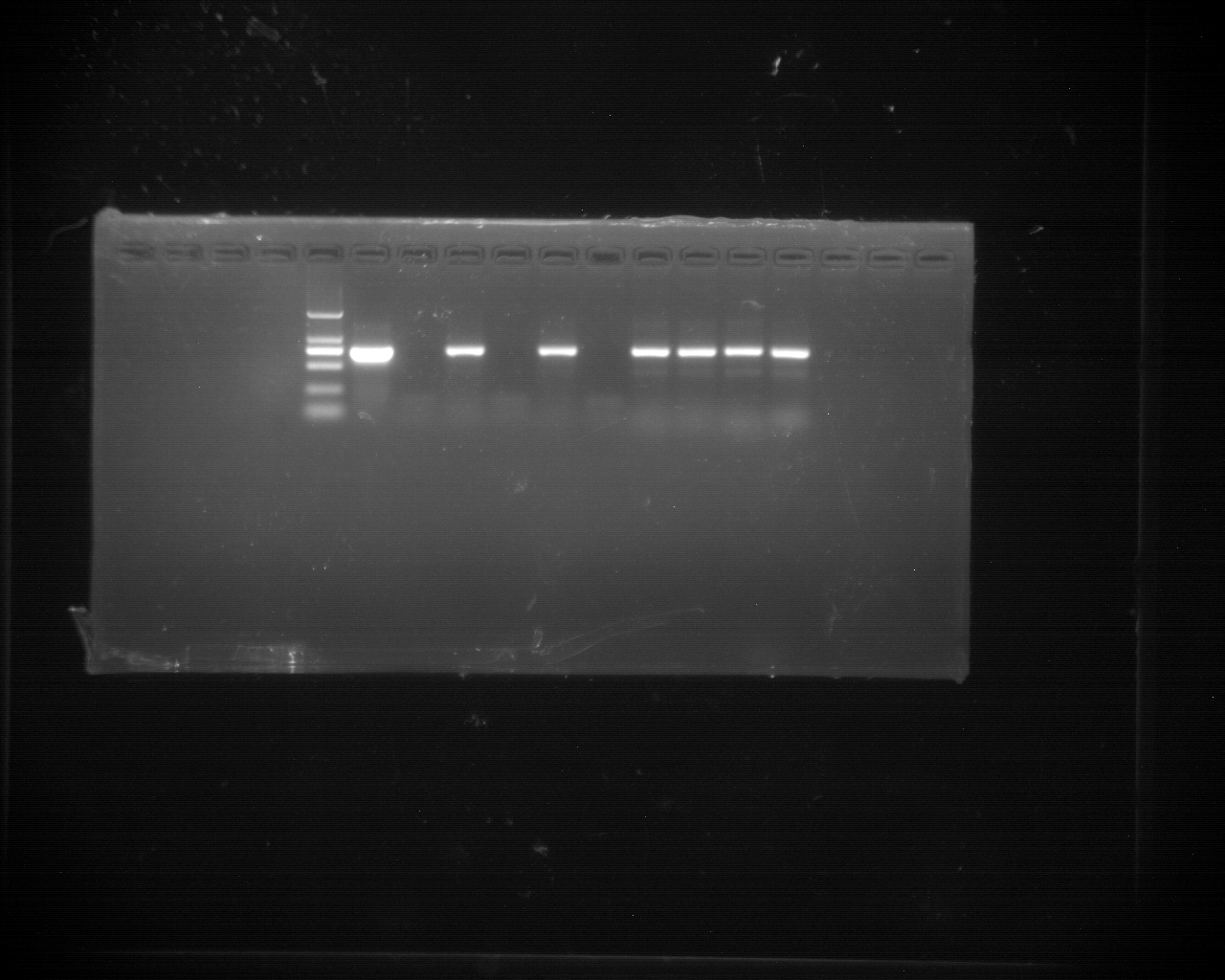

Supplement: Supplementary file 2 — Additional file 2. Transgenic plants were confirmed by PCR. [file 12870_2021_2839_MOESM2_ESM.tif]

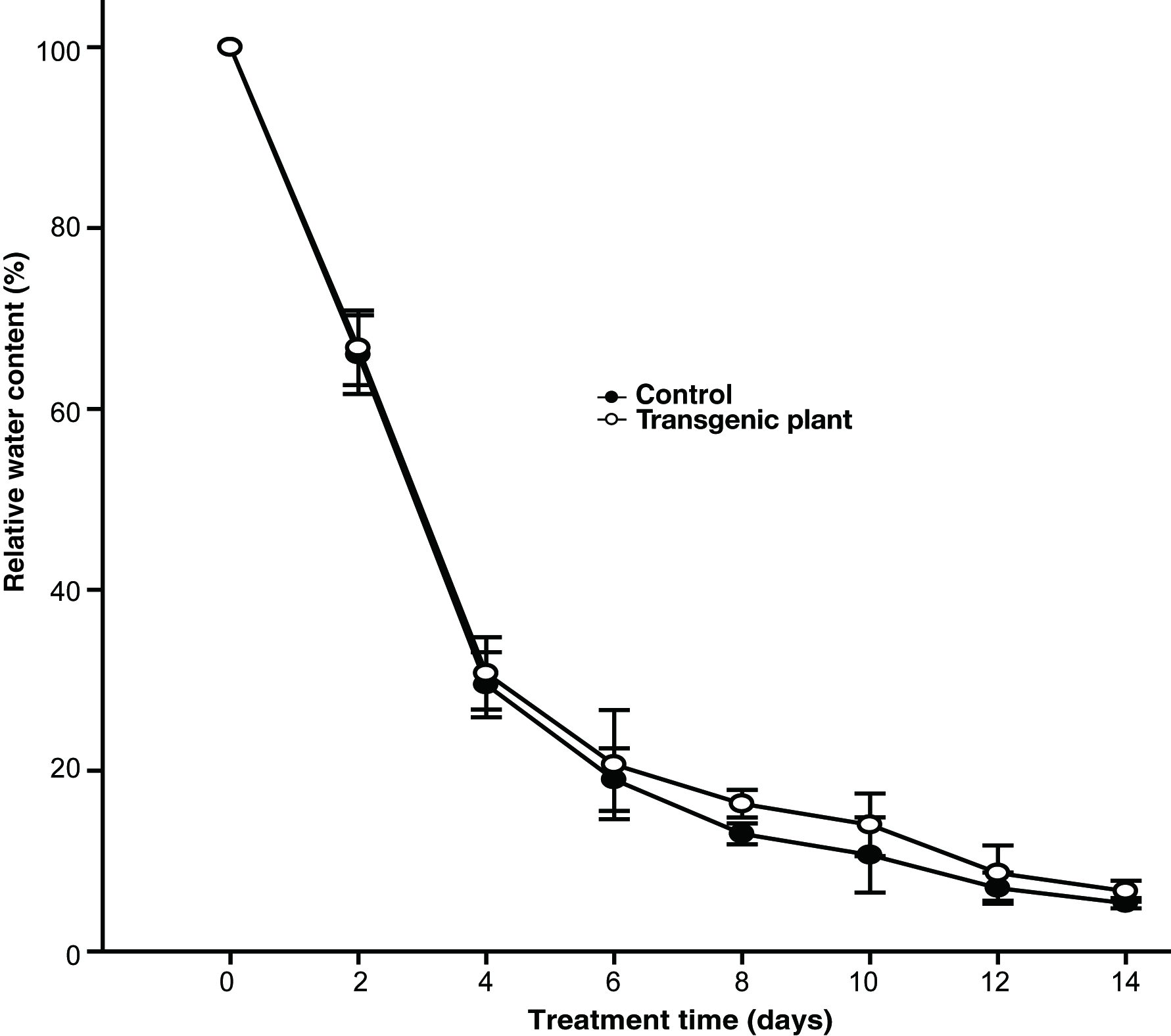

Supplement: Supplementary file 5 — Additional file 5 Relative water content during 0–14 days under drought stress in the control and transgenic plants. Relative water content was measured using the soil temperature/moisture meter every 2d during 0–14 days of drought stress. Three biological replicated were performed. Since the mixed vermiculite and soil was saturated with 0.8 L water, the relative soil water content before drought was set as 100%. Data are presented as the mean ± SD (n = 3). [file 12870_2021_2839_MOESM5_ESM.tif]

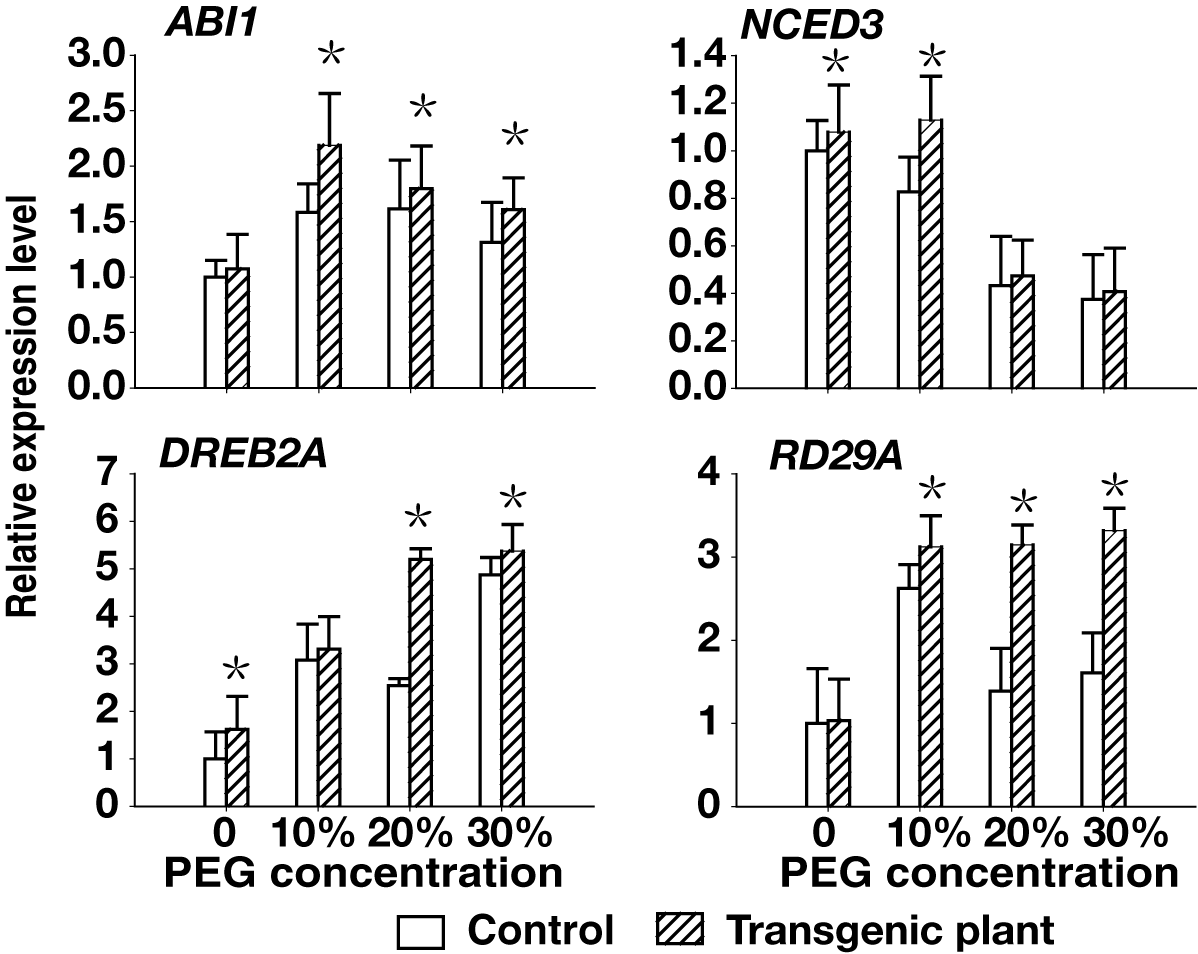

Supplement: Supplementary file 6 — Additional file 6 Relative expression levels assay during the PEG treatment in the control and transgenic plants. Relative expression levels were quantified by a RT-qPCR assay, which used Actin as a control. The 30 days old control and transgenic plants heterologously expressing rty cultured for on MS medium adding 6 - BA 0.2 mg L− 1 and IBA 0.1 mg L− 1 were transferred to 100-mL glass flasks containing 0% (CK), 10, 20%, or 30% PEG-infused medium at 21 °C in a temperature-controlled growth room with a 16-h light/8-h dark photoperiod. Forty-eight hours later, the treated plants were sampled to assay. Three biological replicates were performed per treatment. Data are presented as the mean ± SD (n = 3) (*P < 0.05, Student’s t test). [file 12870_2021_2839_MOESM6_ESM.tif]

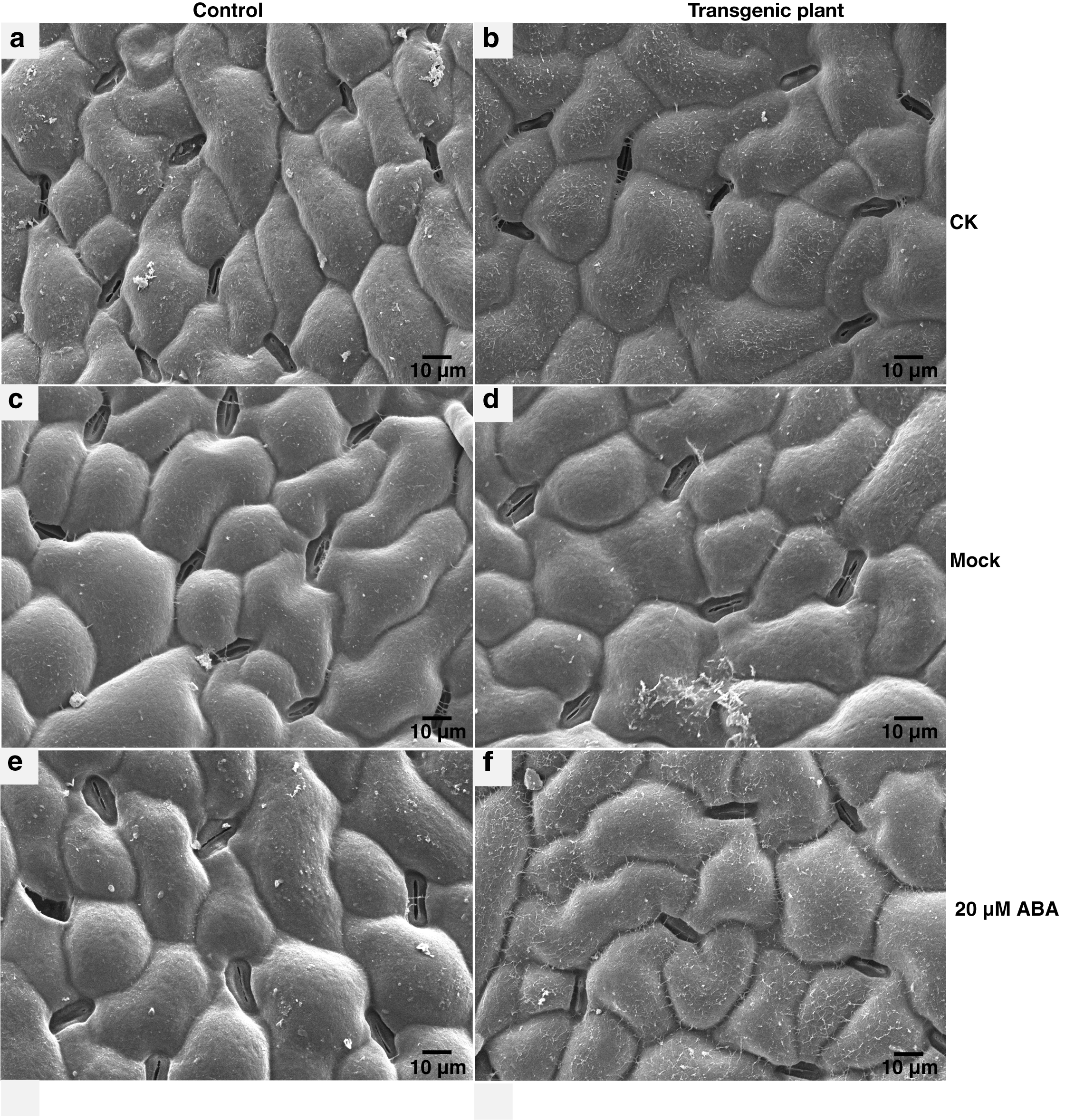

Supplement: Supplementary file 7 — Additional file 7. ABA-induced stomatal closure by scanning electron microscopy in the control and transgenic plants. (a) and (b) control and transgenic plant were no treated with stomatal opening solution and ABA; (c) and (d) control and transgenic plant were treated 2 h with the same volume of ethanol as a control after treatment with stomatal opening solution 2 h; (e) and (f) control and transgenic plant were treated with the 20 μM ABA 2 h after treatment with stomatal opening solution 2 h. [file 12870_2021_2839_MOESM7_ESM.tif]
